# Association between workplace psychological violence and work engagement among emergency nurses: The mediating effect of organizational climate

**DOI:** 10.1371/journal.pone.0268939

**Published:** 2022-06-01

**Authors:** Huiling Hu, Haiyan Gong, Dongmei Ma, Xue Wu

**Affiliations:** 1 Peking University School of Nursing, Beijing, P.R. China; 2 Department of Nursing, China-Japan Friendship Hospital, Beijing, P.R. China; 3 Department of Emergency, Beijing Hospital, National Center of Gerontology, P.R. China; St John’s University, UNITED KINGDOM

## Abstract

**Background:**

Given that increasing attention is being given to the burdens on medical systems, researchers have concentrated their attention on nurses’ work engagement, especially in emergency departments.

**Purpose:**

To investigate the current situation of work engagement of nurses in emergency department, and to find out the impact of psychological violence on work engagement and its impact path.

**Basic procedures:**

The research is a cross-sectional study. Questionnaires were distributed to 243 nurses from the emergency departments of ten tertiary hospitals from September to October 2019. SPSS was used to conducted ANOVA. The AMOS was used to conduct structural equation model to test the mediating effect of organizational climate on the association between psychological violence and dimensions of work engagement.

**Main findings:**

Psychological violence was negatively correlated with organizational climate, vitality, dedication, and focus, and organizational climate was positively correlated with dimensions of work engagement. A negative relationship was found between psychological violence and three dimensions of work engagement, which was mediated by organizational climate.

**Conclusion:**

In order to curb workplace psychological violence and improve the work engagement level of emergency nurses, organizational climate can be used as an intervention measure. The support of leaders, the care of colleagues and the mutual understanding and communication between doctors and patients can alleviate the job burnout of nurses in the face of heavy work, so that nurses can face their daily work with a better mental outlook.

## Introduction

In 2020, the total number of registered nurses in China was 4.71 million [1], with 3 nurses per 1,000 people, far below the OECD (Organization for Economic Cooperation and Development) average of 9 nurses per 1000 [2]. Especially in emergency departments, the increase of the emergency treatment rate per capita and the increase in the aging population, places more and more pressure on nursing staff [3].

Work engagement is a multi-dimensional motivational concept that entails the overall and all-round investment of individuals in their work and the structural connections among personal characteristics, organizational factors, and work performance [4]. Work engagement has an optimal effect on employee performance and organizational outcomes [5, 6]. It has been found that there is a positive correlation between nurses’ work engagement and patient experience, nursing quality and patient safety [7–10]. One unit reduction in work engagement will lead to a 29% reduction in hospital safety level [7]. According data on 4,000 nurses, only 26.0% of emergency nurses are highly engaged in their work [11]. Although this percentage seems slightly high, work engagement is an important factor related to improving patient outcomes, employee retention and job satisfaction. In Portuguese, work engagement is an important and negative predictor of turnover intention among nurses [12]. In Italy and China, work engagement also has a significant negative impact on turnover intention [13, 14]. Among emergency nurses, lack of work engagement is a significant predictor of turnover intention, job burnout and job dissatisfaction [15, 16]. In a word, the role of work engagement is becoming more and more significant by achieving various positive results for organizations and individuals. It can improve job satisfaction [17] and reduce psychological pressure [18]. In nursing service, high level of work engagement reduces turnover intention, work delay and absence [19], and enhances emotional health [20]. On the other hand, work engagement will have an impact on work efficiency, nursing quality and patient satisfaction, which in turn affects organizational results. Therefore, how to improve the work engagement of nursing staff is the responsibility of health managers.

## Background

The definition of work engagement in this study is specific to nursing, based on a concept analysis conducted by Bargagliotti [21], who used the method of conceptual analysis to propose that in the field of nursing, work engagement is a focused, fascinating and dynamic nursing practice. It originates from an independent and trusted environment and can bring safer and more cost-effective patient outcomes. It emphasizes that dynamic care practices emerge from an environment of autonomy and trust, which results in safer and cost-effective patient outcomes. Work engagement is characterized by vitality, dedication and focus. Vitality is manifested in a high level of energy and psychological flexibility at work. Dedication means being fully committed to your work and experiencing a sense of importance and enthusiasm. Focus is considered to be complete concentration and pleasant immersion in one’s work. The predictors of work engagement mainly include organizational antecedents and personal antecedents, among which organizational antecedents include areas of work-life, structural empowerment, work climate and support, leadership, interpersonal relationship, etc [22]. Organizational climate and workplace psychological violence are the organizational antecedents that affect work engagement. In addition, job demand resource (JD-R) model is one of the most commonly used theories to study employees’ work engagement in organizational context [23]. JD-R model holds that individual work engagement is the result of a large number of work resources provided by organizations and people’s own resources. Work resources are physiological, psychological, social and organizational characteristics that can help individuals effectively face various work requirements, and can promote personal and career development [24]. Organizational climate and psychological violence are important individual resources.

Studies have shown that workplace violence is more frequent in emergency and pediatric departments in both developing and developed countries [25, 26]. Extensive survey data show that more than half of nurses in many countries have experienced workplace violence—Turkey 51% [27], Australia 67% [28] and China 68% [29]. Among all the emergency nurses who experienced workplace violence, 61.0% had the intention to leave [30]. Workplace violence refers to verbal assaults, threats, abuse, and even physical attacks from colleagues and team workers during the process of work, resulting in psychological or/and physical injuries [31]. In clinical work, workplace psychological violence is more common than physical violence [32]. The incidence of workplace psychological violence is three times higher against nurses than it is against other medical personnel [33]. Also, emergency nurses are more likely to experience workplace violence than nurses in other departments, of which verbal violence accounts for 64.3% [34], and almost 70% of the nurses in one survey suffered verbal abuse in the workplace [35]. This shows that in the hospital environment, emergency nurses are the group most affected by psychological violence in the workplace. Psychological violence in the workplace seriously affects the physical and mental health of nurses [36]. Although workplace psychological violence does not leave scars, the emotional harm to the victims can be destructive, including strong emotional reactions, psychological discomfort, increased attrition, career changes and decreased quality of care provided by nurses [37]. It is an important reason for the reduced work engagement of employees [38]. Leymann defined workplace psychological violence as “a kind of psychological terror, which is manifested as systematic, direct, immoral communication and hostile behavior of one or more individuals to an individual” [39]. In our study, workplace psychological violence refers to the damage to physical, mental, spiritual, moral and social development caused by deliberately opposing others or the collective, including verbal abuse, insults, threats, attacks, torture and verbal harassment [40]. As we are concerned about psychological violence among organizations, workplace psychological violence, this research was initiated by leaders, coworkers, subordinates and other medical staff.

The existing literature provides evidence that certain domain-specific climates are related to work engagement, such as a social climate [41] and supporting climate [42]. A study of medical staff, which regarded organizational climate as a kind of work resource, found it had a positive correlation with work engagement [43]. This is also consistent with the JD-R model mentioned above. Organizational climate refers to employees’ perceptions of organizational management, such as decision-making and standardization of work units [44]. A 2013 study also found that the organizational climate within a team of workers had a positive effect on work-related behaviors, attitudes, and efficiency [45]. A good organizational climate can improve employees’ sense of belonging, and hence, make them more reluctant to leave the organization [44]. It is very important to distinguish organizational climate from organizational culture. Because they are more similar in structure, and the concept of organizational culture is more extensive than the concept of organizational climate. Organizational culture and organizational climate are closely related concepts. They both refer to the way that members of an organization understand the environment, and they all represent the common connotation that constitutes the basis of behavior. Culture is a deeper level of the organization, while climate is the visible daily life of the organization. Therefore, some members may not be able to fully experience the cultural aspect of the organization (that is, deep values), but all members of the organization can experience the climate of the organization (that is, the perception of the environment) [46].

There are only a few studies on the relationship between workplace psychological violence and organizational climate. A survey among university officials found that workplace psychological violence was negatively correlated with organizational climate [47], and a survey of nurses found that bullying among nurses was also negatively correlated with organizational climate [48]. Therefore, we assume that workplace psychological violence is negatively correlated with organizational climate.

Workplace psychological violence has been shown to have a negative association with work engagement, whereas organizational climate has a positive association with work engagement. However, the relationships among these three variables are unclear. As far as we know, organizational climate has not been clearly demonstrated to be a mediator between psychological violence and work engagement, especially among emergency nurses. Therefore, we hoped to add organizational climate into the work engagement model of emergency nurses. The purpose of this study was to explore the mediating role of organizational climate, hoping to provide a basis for improving the work engagement of emergency nurses from another angle.

The hypotheses of this study were as follows.

*Hypothesis 1*: Psychological violence is negatively correlated with organizational climate and dimensions of work engagement;

*Hypothesis 2*: Organizational climate is positively correlated with dimensions of work engagement;

*Hypothesis 3*: There is an inverse relationship between psychological violence in the workplace and dimensions of work engagement, which is mediated by organizational climate.

## Methods

The research was approved by Peking University Institutional Review Board; the approval number is IRB00001052-19047; the form of consent obtained was by written.

### Aims

The aim was to understand the status of work engagement, psychological violence, and organizational climate among nurses working in emergency departments by exploring the relationships among these three variables and to test the mediating effect of organizational climate.

### Design

This was a cross-sectional study conducted in Beijing, China, between September and October 2019.

### Participants

We selected ten tertiary hospitals by convenient sampling in Beijing and a sample of 243 Registered Nurses working in emergency departments. The inclusion criteria were holding a nurse qualification certificate that was issued within the registration time limit, working in an emergency department for more than 6 months, and agreeing to participate voluntarily. The exclusion criteria were nurses engaged in advanced studies in the hospitals, head nurses, workers who were off duty due to illness or compassionate leave, and workers who were unavailable because they were on leave from the hospital for educational or other reasons. We set up screening questions before filling in the questionnaire to identify the sample, such as “whether you hold the nurse qualification certificate”, “working years”, “position”, etc. We calculated the sample size to be 5–10 times of the total number of items (37 items, NOCS) and assuming a 20% loss to follow-up. The required sample size was at least 222, so we used a sample size of 243 nurses. It also conforms to the principle that at least 200 samples are required for structural equation modeling [49]. G-Power software was also used to calculate the sample size of ANOVA and correlation analysis. When the *“Correlation*: *Point biserial model”* was choose as the statistical test and input parameters were set as effect size ρ = 0.3 and α = 0.05, the total sample size was 134. When the *“ANOVA*: *Fixed effects*, *omnibus*, *one-way”* was choose as the statistical test and input parameters were set as effect size f = 0.25, α = 0.05 and number of groups = 5, the total sample size was 200, which also supports the rationality of the sample size in this survey.

### Data collection

A contact person from the hospital was assigned to be responsible for distributing links to the questionnaire and collecting the questionnaire data. The electronic questionnaire was distributed from September to October 2019, using “Questionnaire Star,” which is a professional platform for collecting survey data. We also collected data on the participants’ age, sex, years worked in an emergency department, highest educational qualification, academic title, personnel appointment, marital status, monthly income and frequency of overtime work. There was a statement at the beginning of questionnaire, which explained informed consent was given if you completed the questionnaire. Additionally, the questionnaire can only be submitted after all the items are completed. Consequently, altogether 243 nurses finished the online survey and every questionnaire was valid.

### Variables and instruments

#### Workplace psychological violence

Workplace psychological violence was measured using the Workplace Psychologically Violent Behaviors instrument (WPVB), which was developed by Dilek and Aytolan [50] and translated and revised by Xu et al. [51]. This scale is composed of 4 dimensions and 32 items: isolated at work (11 items), attacks on professional status (9 items), attacks on personality (9 items), and direct negative behavior (3 items). The items are rated on a scale of 0 to 5: 0 (never), 1 (rarely), 2 (sometimes), 3 (occasionally), 4 (often), and 5 (always). The total score ranges from 0 to 160, with higher scores indicating a higher frequency of suffering workplace psychological violence. The Cronbach’s α coefficient is 0.96, and the α coefficients for the dimensions are between 0.85 and 0.94 [42].

#### Organizational climate

Nurses’ perceptions of organizational climate were measured using the Nurse’s Organizational Climate Scale (NOCS), which was developed by He, Hou, and Cao [52], based on the Stone et al. [44] framework for the Integrative Model of Organizational Climate (IMOC). The NOCS consists of 6 dimensions and 37 items, which include 10 items on resource assurance, 8 items on team behavior, 9 items on management support, 4 items on quality management, 4 items on human resource management, and 2 items on evidence-based nursing support. The NOCS uses a 4-point Likert scale: 1 (strongly disagree), 2 (disagree), 3 (agree), and 4 (strongly agree). The total score ranges from 37 to 148 points, with higher scores indicating a better organizational climate. The Cronbach’s α for the NOCS was 0.94, and the Cronbach’s α for each dimension was between 0.75 and 0.88 [53].

#### Work engagement

Work engagement was measured using the Chinese version of the Utrecht Work Engagement Scale (UWES) [54] that was developed by Zhang and Gan [55]. The UWES consists of three dimensions–vitality (6 items), dedication (4 items), and focus (5 items)–and uses a 7-point rating scale ranging from 0 (never) to 6 (always). A score of more than 4 points is defined as a high level of work engagement [55, 56]. The range of the total score is 0 to 90 points. Higher scores indicate higher work engagement. The Cronbach’s α for the UWES is 0.90, and the Cronbach’s α for the three dimensions are 0.77 (vitality), 0.74 (dedication) and 0.75 (focus) [55].

#### Ethical considerations

Ethics committee approval of this study was obtained from the university where the corresponding author works.

#### Statistical analysis

SPSS 22.0 (IBM Corp. Armonk, NY) was used for the statistical analyses. All statistical tests are two-way (α = 0.05). The *t*-test was used to compare differences in the outcome measures for demographic characteristics that were classified into two categories and Analysis of Variance (ANOVA) was used to compare differences in the outcome measures for demographic characteristics that were classified into three or more categories, such as age, working years in emergency, monthly income, title and overtime working. Gender, title and overtime working were used as control variables to conduct partial correlation, which explore the correlation among workplace psychological violence, organizational climate and the dimensions of work engagement. Amos 24.0 was used to draw a hypothesis model, with workplace psychological violence as the independent variable, organizational climate as the mediating variable, and three dimensions of work engagement the dependent variable. The maximum likelihood method was used to calculate. If the 95% confidence intervals do not include 0, the indirect effect (mediation) is statistically significant. If an mediating relationship was established, the following conditions should be satisfied [57]: 1) The independent variable (workplace psychological violence) is significantly associated with the dependent variable (three dimensions of work engagement), 2) the independent variable (workplace psychological violence) is significantly associated with the mediator (organization climate), and 3) the mediator (organization climate) is significantly associated with the dependent variable (three dimensions of work engagement), and the effect of the independent variable (workplace psychological violence) on the dependent variable (three dimensions of work engagement) reduces when the mediator (organization climate) is added to the model (partial mediator). If the independent variable does not affect the dependent variable when the mediator is added to the model, then full mediation is established.

## Results

### Characteristics of the participants

The 243 people who completed the questionnaire served as the participants in the study. The mean age and mean years of work of the sample were 33.0 years and 10.7 years, respectively; 89.7% of the participants were women. The total scores of the psychological violence, work engagement, and organizational climate scales are shown in Table 1, along with the scores for each of their dimensions. The mean total score for organizational climate (108.6 points) was in the middle of the range of possible scores (37–185 points) and the mean team behavior score (2.7 points) was the lowest of the NOCS’s six dimensions. The mean total score for psychological violence (63.3 points) was also in the middle range of possible scores (0–160 points), with the mean score for direct negative behavior being the lowest score (1.6 points) of the WPVP’s four dimensions. The mean total score for work engagement (65.8 points) was somewhat closer to the higher end of the scale’s range (0–90 points), with the mean score for dedication (4.2 points) being the lowest of the UWES’s three dimensions.

**Table 1:** The score of psychological violence, organizational climate, work engagement.

Categories	Number of items(total scores)	Score [mean (SD)]	Item equipartition
**Organizational climate**	**37(148)**	108.6(20.4)	2.9(0.2)
Resource assurance	10(50)	29.1(6.0)	2.9(0.6)
Team behavior	8(40)	22.0(5.2)	2.7(0.7)
Management support	9(45)	27.0(5.0)	3.0(0.5)
Quality management	4(20)	12.3(2.1)	3.1(0.5)
Human resource management	4(20)	12.2(2.2)	3.1(0.6)
Evidence-based nursing support	2(10)	6.0(1.3)	3.0(0.7)
**Psychological violence**	**32(160)**	63.3(27.4)	2.0(0.3)
Isolated in work	11(55)	23.3(10.4)	2.1(0.9)
Attacking professional status	9(45)	18.7(9.1)	2.1(1.0)
Personality attack	9(45)	16.8(7.7)	1.8(0.9)
Direct negative behavior	3(15)	4.8(2.5)	1.6(0.8)
**Work engagement**	**15(90)**	65.8(18.0)	4.6(0.4)
Vitality	6(36)	27.5(7.7)	4.6(1.3)
Dedication	4(24)	16.8(5.3)	4.2(1.3)
Focus	5(30)	21.5(6.3)	4.3(1.2)

Table 2 shows the demographic characteristics of the sample and the three outcome measures by demographic characteristics. Table 2 shows that overtime working was related to three dimensions of work engagement and organizational climate score (*F* = 5.159, *p* = 0.001). More frequent overtime working was associated with the lower work engagement. The title of nurses was related to three dimensions of work engagement. Therefore, overtime working and title were included as control variables in SEM to exam the mediating effect.

**Table 2:** The demographic characteristics.

Categories	Number (%)	Organizational climate (Score, mean (SD))	Workplace psychological Violence (Score, mean (SD))	Work engagement (score, mean (SD))		
				Vitality	Dedication	Focus
**Age**						
20–30	104(43.2%)	109.0(19.9)	61.4(27.4)	26.7(7.1)	16.3(5.1)	20.9(5.6)
31–40	100(41.2%)	107.6(22.3)	64.3(27.5)	28.0(8.0)	17.1(5.4)	21.7(6.9)
41–50	33(13.6%)	108.6(16.4)	67.5(25.7)	28.2(7.5)	17.1(5.0)	22.2(6.1)
>50	5(2.1%)	115.8(17.4)	62.0(38.3)	29.8(14.4)	19.6(8.2)	25.0(7.3)
*F*		0.297	0.470	0.766	0.911	0.960
**Gender**						
Female	218(89.7%)	107.8(20.0)	61.7(26.0)	27.3(7.7)	16.7(5.2)	21.5(6.3)
Male	25(10.3%)	115.4(22.7)	77.3(35.1)	29.5(7.4)	17.3(6.3)	22.0(6.3)
*t*		1.789	2.731*	1.340	0.481	0.402
**Marital status**						
Unmarried	63(25.9%)	109.3(19.4)	58.2(26.6)	26.6(6.4)	16.5(4.5)	20.9(4.6)
Married/Divorce	180(74.1%)	108.3(20.7)	65.1(27.5)	27.8(8.1)	16.9(5.5)	21.7(6.7)
*t*		0.317	-1.712	-1.060	-0.562	-0.933
**Working years in emergency (years)**						
<5	50(20.6%)	108.5(22.7)	59.1(25.1)	27.3(6.8)	16.5(4.8)	20.9(5.0)
5–15	127(52.3%)	107.0(20.3)	64.4(28.0)	27.4(7.6)	16.7(5.6)	21.6(6.8)
15–25	52(21.4%)	112.2(19.9)	64.3(28.8)	27.1(9.2)	16.8(5.2)	21.4(6.4)
≥25	14(5.8%)	109.5(12.8)	64.9(25.2)	30.9(5.9)	18.7(4.1)	23.3(5.1)
*F*		0.799	0.502	0.975	0.667	0.545
**Monthly income (yuan)**						
<5000	40(16.5%)	112.3(26.3)	58.5(27.3)	28.6(9.1)	17.8(6.7)	23.2(8.3)
5000~7999	47(19.3%)	107.4(18.5)	64.8(29.1)	28.6(7.7)	17.5(5.5)	22.5(6.5)
8000~10999	83(34.2%)	108.4(17.8)	60.8(27.7)	26.7(6.9)	16.0(4.8)	20.2(5.1)
11000~14999	53(21.8%)	107.4(20.3)	64.9(23.7)	26.7(8.2)	16.8(4.4)	21.4(5.7)
>15000	20(8.2%)	107.5(22.3)	75.9(29.5)	28.2(7.0)	16.2(5.5)	21.7(6.3)
*F*		0.423	1.636	0.856	1.079	1.934
**Title**						
Junior	135(55.6%)	110.1(22.5)	61.5(29.0)	27.6(8.1)	17.0(5.6)	21.6(6.4)
Intermediate	100(41.2%)	106.6(17.4)	65.6(25.4)	26.9(7.2)	16.3(4.7)	20.9(5.9)
Senior	8(3.3%)	108.5(15.9)	64.8(24.6)	34.1(3.3)	21.0(4.0)	28.0(4.3)
*F*		0.847	0.650	3.354*	3.188*	4.963*
**Highest education qualification**						
Junior college and below	55(22.6%)	112.6(23.0)	59.8(27.7)	26.9(8.4)	16.5(5.7)	20.9(6.0)
Undergraduate and above	188(77.4%)	107.4(19.4)	64.3(27.3)	27.7(7.5)	16.9(5.2)	21.7(6.3)
*t*		1.691	-1.089	-0.649	-0.490	-0.875
**Overtime working**						
Never	8(3.3%)	120.3(14.5)	58.8(31.1)	29.9(10.8)	18.1(6.8)	22.4(8.3)
Occasionally	90(37.0%)	112.6(18.3)	59.4(26.8)	28.7(7.8)	17.7(5.5)	22.6(5.8)
Sometimes	59(24.3%)	110.0(20.7)	64.4(29.0)	27.8(7.5)	16.9(5.3)	21.7(6.7)
Often	75(30.9%)	104.3(20.2)	67.4(27.0)	26.5(7.2)	16.3(4.4)	20.9(5.6)
Always	11(4.5%)	89.6(24.5)	64.7(23.4)	21.7(7.0)	11.3(5.0)	15.7(7.4)
F		5.159*	0.964	2.669*	4.123*	3.406*

*p < 0.05

### The relationships among workplace psychological violence, organizational climate and work engagement (H1, H2)

The partial correlation analysis revealed that psychological violence was negatively correlated with organizational climate (*r* = -.448, *p* < 0.001) and three dimensions of work engagement, whereas organizational climate was positively correlated with three dimensions of work engagement (Table 3). The above results confirmed Hypotheses 1 and 2.

**Table 3:** Partial correlation results of each variable.

	Vitality	Dedication	Focus	Organizational climate
**Organizational climate**	0.536**	0.489**	0.476**	
**Workplace psychological Violence**	-0.331**	-0.256**	-0.192**	-0.448**

**p<0.001

### Organizational climate mediates the relationship between workplace psychological violence and work engagement (H3)

**Fig 1:**
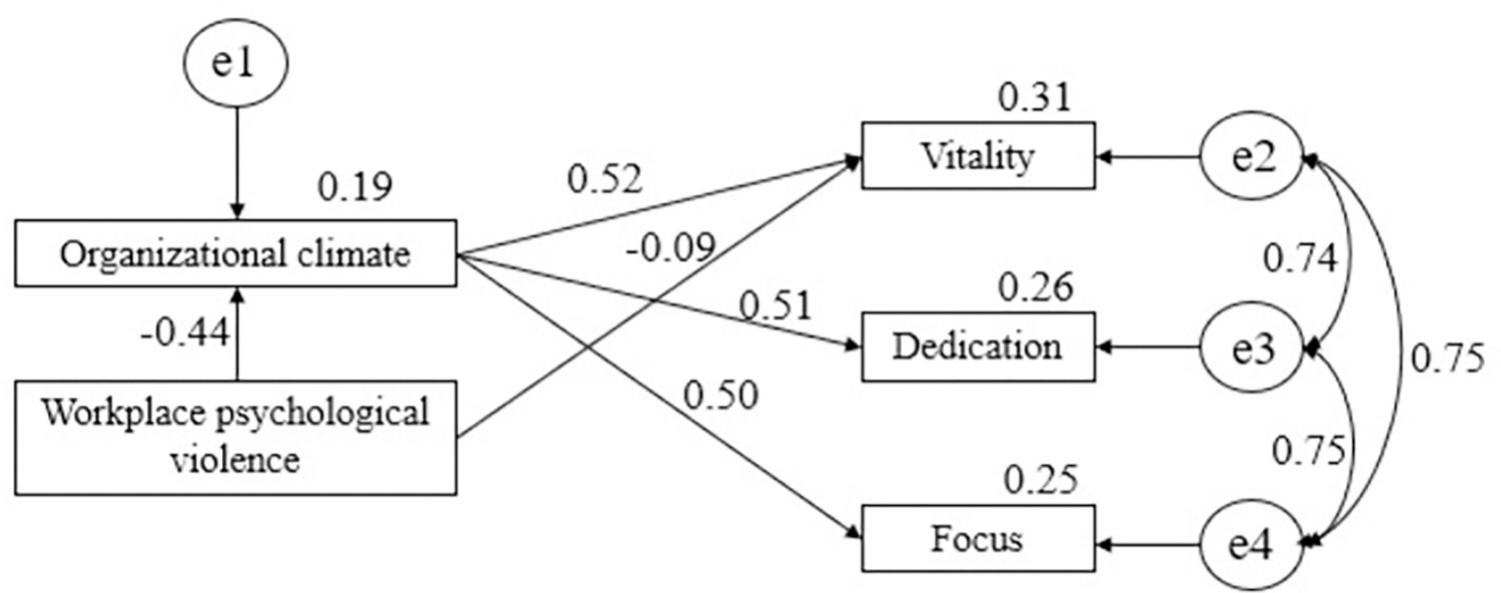
Organizational climate mediation models of the relationship between psychological violence and dimensions of work engagement (standardized).

The overall fitness indexes of the structural equation model were CMIN/df 1.332, RMSEA 0.037, SRMR 0.0112, GFI 0.996, IFI 0.999, CFI 0.999 and TLI 0.995. It shows that the model has a good overall fitness. As shown in Fig 1, workplace psychological violence has significant effect on all dimensions of work engagement. After controlling the title and overtime working that have a significant impact on the dimensions of work engagement, organizational climate mediates the relationship between workplace psychological violence and dimensions of work engagement. A negative relationship was found between psychological violence and three dimensions of work engagement (*β*_*1*_ = -.312, *β*_*2*_ = -.224, *β*_*3*_ = -.217), which was mediated by organizational climate (*β*_*4*_ = -.226, *β*_*5*_ = -.224, *β*_*6*_ = -.217). Workplace psychological violence only has a significant direct effect on the dimension of vitality (*β*_*7*_ = -.086). See Table 4 and the results confirmed Hypothesis 3.

**Table 4:** Total effects, direct effects, and indirect effects of workplace psychological violence and organizational climate on work engagement.

	B	β	SE	95%CI
***Total effects***				
Workplace psychological violence→organizational climate	-0.325	-0.437	0.055	(-0.542, -0.330)
Workplace psychological violence→vitality	-0.088	-0.312	0.041	(-0.393, -0.232)
Workplace psychological violence→dedication	-0.043	-0.224	0.041	(-0.310, -0.147)
Workplace psychological violence→focus	-0.050	-0.217	0.040	(-0.300, -0.142)
Organizational climate→vitality	0.196	0.496	0.064	(0.385, 0.640)
Organizational climate→dedication	0.133	0.512	0.058	(0.391, 0.616)
Organizational climate→focus	0.152	0.518	0.063	(0.385, 0.607)
***Direct effects***				
Workplace psychological violence→vitality	-0.024	-0.086	0.040	(-0.168, -0.009)
***Indirect effects***				
Workplace psychological violence→vitality	-0.064	-0.226	0.045	(-0.325, -0.148)
Workplace psychological violence→dedication	-0.043	-0.224	0.041	(-0.310, -0.147)
Workplace psychological violence→focus	-0.050	-0.217	0.040	(-0.325, -0.148)

## Discussion

Through the mediation analysis, we found that: 1) Workplace psychological violence is negatively correlated with organizational climate and dimensions of work engagement; 2) Organizational climate is positively correlated with dimensions of work engagement; and 3) There is an inverse relationship between workplace psychological violence and dimensions of work engagement, which is mediated by organizational climate. The following sections will discuss the scores of the three variables, respectively, and the mediating role of organizational climate.

### The emergency nurses have relatively high levels of work engagement

The results of this study show that the average score of the work engagement of nurses in emergency departments is relatively high, which is similar to the results of other studies [56, 58]. We also found the frequency of overtime affected work engagement, which is similar to the results of Watanabe and Yamauchi [59]. Of the 243 nurses, 58.4% were at a high level of work engagement, which indicates that nursing managers need to pay attention to the current level of work engagement of clinical nurses, especially in the case of a manpower shortage. They also must stimulate nurses’ work enthusiasm, improve work efficiency, and guarantee nursing quality through scientific management. The key to nurse’s work engagement is to have meaningful work that enables nurses to live their values [60].

### The emergency nurses have relatively high levels of workplace psychological violence

The results of this study show that the level of workplace psychological violence in emergency nurses is higher than that in other departments (operating room: 63.3 vs. 30.59, *p*<0.05, inpatient ward: 63.3 vs. 24.17, *p*<0.05) [61, 62]. The reason probably is for this may be that the participants in this study all worked in third-grade hospitals, which have many patients. Emergency departments are notoriously chaotic, unpredictable environments that expose nurses to a wider range of stressors than other nursing units do [63]. Emergency departments often challenge nurses with heavy workloads, have a high risk of exposure to interpersonal conflict and violence and involve high acuity patients [64]. Nurse managers should pay attention to the phenomenon of psychological violence against nurses in the workplace and take targeted measures to create a good relationship, such as conducting team communication training to improve the interpersonal communication ability of nurses, regular communication, and standardize management [65]. In addition, preventive measures and relevant norms of behavior can effectively prevent psychological violence against nurses [66]. The Chinese healthcare system has set an example by adopting a “safe hospital” policy, which uses social media to promote a positive image of nurses and help people understand their contributions [67].

Additionally, we found a negative correlation between workplace psychological violence and work engagement, which means the higher the level of psychological violence, the lower the level of nurses’ work engagement. Therefore, hospital managers should take active and effective measures to improve nurses’ sense of belonging to the hospital and increase their level of work engagement. The factors that affected the frequency of psychological violence in this study were gender and having the highest level of education, which is consistent with the results of Xiao-duo et al. [68].

Further analysis of the scores on all the dimensions showed that the highest scores were for being isolated at work and attacks on one’s professional status, followed by personality attacks. The score on the direct negative behavior dimension was the lowest. The reason for these findings may be that being isolated at work and attacks on one’s professional status are more likely to be implicit attacks, whereas personality attacks and direct negative behavior are more likely to entail explicit violence. Research has shown that implicit aggression is more common in the nursing workplace than explicit aggression [69].

### The emergency nurses perceived at medium level of organizational climate

This study showed that the nurses’ perception of the organizational climate in emergency departments was at a medium level, which is consistent with the results of He et al. [52]. Hospital managers should provide nursing staff with work support and work security by offering various types of professional and technical training and implementing an effective performance appraisal mechanism to create a good organizational climate, which can stimulate the enthusiasm of nursing staff, and thus, affect their degree of work engagement.

The organizational climate of the participants was positively correlated with their total score for work engagement, which is consistent with the results of Aly [70]. Getting the organization’s recognition and affirmation motivates employees to continue to work for the organization and constantly improve themselves. Nurses’ work engagement is not only reflected in their professional skills, but also in their humanistic care and emotional commitment to patients. Nurses with a higher sense of organizational climate should be more fully engaged in their work.

### The mediating role of organizational climate

The results of this study demonstrate the mediating effect of organizational climate on the relationship between workplace psychological violence and work engagement. Especially during the COVID-19 pandemic, nurses faced the problems of large numbers of patients and shortages of staff, and they need to make sure that they have enough work engagement to deal with this kind of emergency [71]. Work engagement is a positive working state of employees, which is an important predictor of job satisfaction and the turnover intention of nurses [72]. Moreover, the work engagement of nurses directly affects their work performance and service quality [73]. Nurse managers should pay attention to building a fair, harmonious, and positive organizational atmosphere to reduce the pressure on nurses, reduce personal negative emotions, and stimulate the work engagement of nurses, so that they can productively and efficiently perform high-quality care.

### Limitations

Our study has some limitations. First, our sample was from hospitals in Beijing, the capital of China, which is densely populated. Given potential regional and individual differences, participants from more regions should be included in future studies to confirm the research results and increase their generalizability. Second, the present study was exploratory and the use of a cross-sectional design does not allow us to infer causality. Although many studies have provided theoretical and empirical support for cross-sectional design [74–76], the hypothesis that there are causal relationships among workplace psychological violence, organizational climate and work engagement needs to be tested by longitudinal studies. Finally, there may be reaction bias due to concerns that participants may answer questions from a biased perspective. They may answer in some way to protect their position. There is a risk of response bias in all surveys because respondents answer according to their own interpretation of the question.

## Conclusion

In summary, this study provides further support for establishing the relationship between workplace psychological violence and work engagement. More importantly, organizational climate predicts work engagement and mediates the relationship between workplace psychological violence and work engagement. Therefore, it can be concluded that in order to curb workplace psychological violence and improve the work engagement level of emergency nurses, organizational climate can be used as an intervention measure. According to the results of this study, the best organizational climate seems to be not only that the workload is lighter, the teamwork is the best, and professional nurses have defined and accepted their clear roles, but also the need to reduce psychological violence in the workplace. Leadership decisions that create this climate can promote nurses’ performance in basic nursing practice and help ensure safe, patient-centered services.

## Supporting information

S1 File Data set of this study. (SAV)
